# Wnt and Hedgehog Signaling Regulate the Differentiation of F9 Cells into Extraembryonic Endoderm

**DOI:** 10.3389/fcell.2017.00093

**Published:** 2017-10-25

**Authors:** Gurjoth S. J. Deol, Tina N. Cuthbert, Mohamed I. Gatie, Danielle M. Spice, Lindsay R. Hilton, Gregory M. Kelly

**Affiliations:** ^1^Molecular Genetics Unit, Department of Biology, University of Western Ontario, London, ON, Canada; ^2^Child Health Research Institute, London, ON, Canada; ^3^Ontario Institute for Regenerative Medicine, Toronto, ON, Canada

**Keywords:** Wnt, Hedgehog, retinoic acid, extraembryonic endoderm formation

## Abstract

Mouse F9 cells differentiate into primitive extraembryonic endoderm (PrE) when treated with retinoic acid (RA), and this is accompanied by an up-regulation of *Gata6*. The role of the GATA6 network in PrE differentiation is known, and we have shown it directly activates *Wnt6*. Canonical Wnt/β-catenin signaling is required by F9 cells to differentiate to PrE, and this, like most developmental processes, requires input from one or more additional pathways. We found both RA and *Gata6* overexpression, can induce the expression of Indian Hedgehog (*Ihh*) and a subset of its target genes through Gli activation during PrE induction. Chemical activation of the Hh pathway using a Smoothened agonist (SAG) also increased Gli reporter activity, and as expected, when Hh signaling was blocked with a Smoothened antagonist, cyclopamine, this RA-induced reporter activity was reduced. Interestingly, SAG alone failed to induce markers of PrE differentiation, and had no effect on Wnt/β-catenin-dependent TCF-LEF reporter activity. The expected increase in Wnt/β-catenin-dependent TCF-LEF reporter activity and PrE markers induced by RA was, however, blocked by cyclopamine. Finally, inhibiting GSK3 activity with BIO increased both TCF-LEF and Gli reporter activities. Together, we demonstrate the involvement of Hh signaling in the RA-induced differentiation of F9 cells into PrE, and while the activation of the Hh pathway itself is not sufficient, it as well as active Wnt/β-catenin are necessary for F9 cell differentiation.

## Introduction

The mouse blastocyst is comprised of three cell types in preparation for implantation: (1) trophectoderm; (2) pluripotent cells of the inner cell mass; (3) and primitive endoderm (PrE), the initial cell type in the extraembryonic endoderm (ExE) lineage (Kelly and Drysdale, 2015). Elucidating the differentiation of these lineages is difficult to study *in vivo*, and for that reason there are alternative *in vitro* models including the F9 teratocarcinoma cell line that can be chemically induced by retinoic acid (RA) to differentiate into ExE-like cell types (Kelly and Gatie, 2017). Although much is known regarding the differentiation of F9 cells into ExE lineages, an understanding of the signaling mechanism(s) is far from complete. Previous work has shown that Wnt signal transduction pathways are involved in the process (Liu et al., 1999; Bikkavilli et al., 2008; Hwang and Kelly, 2012), and these are initiated by GATA6, a master regulator of endoderm and extraembryonic endoderm formation (Hwang and Kelly, 2012; Kelly and Drysdale, 2015). Wnt signaling plays an integral role in many vertebrate and invertebrate developmental events, specifically in regards to cell proliferation, cell survival, cell behavior, and cell fate decisions in embryos and adults (Moon, 2004; Willert and Nusse, 2012). WNT ligands signal in at least three different pathways, and in the case of the canonical β-catenin pathway, activation begins when WNT binds to a Frizzled receptor and LRP5/6. This interaction recruits Disheveled to the plasma membrane thereby disabling a β-catenin destruction complex comprised of AXIN, adenomatous polyposis coli, casein kinase 1-α, and glycogen synthase kinase3β (GSK3β; Clevers et al., 2014; Cruciat, 2014). With the disassembly of the destruction complex, β-catenin accumulates in the cytoplasm and eventually translocates into the nucleus where it interacts with the lymphoid-enhancing factors (LEF) and T-cell factors (TCF) to initiate the transcription of target genes (Moon, 2004). RA signaling in F9 cells increases WNT6 activity, which leads to the stabilization of β-catenin, and in conjunction with TCF-LEF, leads to the regulation of genes required for PrE formation (Krawetz and Kelly, 2009). Although, these results underpin the importance of Wnt signaling in PrE differentiation, other pathways including Hedgehog (Hh) are also involved (Becker et al., 1997).

Hh is a morphogen that plays a major role in tissue and organ development in invertebrate and vertebrate species (Briscoe and Small, 2015; Jia et al., 2015; Tickle, 2015; Ingham, 2016). In mammals there are three Hh genes, Sonic (*Shh*); Indian (*Ihh*); and Desert (*Dhh*), which encode ligands that signal in a canonical pathway reminiscent to that described for WNTs (Kalderon, 2002; Nusse, 2003). The Hh pathway consists primarily of a series of repressive interactions, beginning with the Hh receptor Patched (PTCH; Ingham and McMahon, 2001), which represses Hh target gene expression by inhibiting Smoothened (SMO; Hooper and Scott, 2005). When Hh is present, however, SMO is active, allowing GLI transcription factor activity to up-regulate Hh-specific target genes (Hui and Angers, 2011; Sasai and Briscoe, 2012). *In vitro* studies showing F9 cells treated with RA show increased *Ihh* expression would suggest that this signaling pathway also plays a role in ExE differentiation in the mouse embryo (Becker et al., 1997), and *in silico* analysis from our lab has identified a putative binding site for GATA6 in the *Ihh* promoter (Supplementary Figure 1). A link between GATA6 and Hh exists, and while IHH rescues definitive hematopoiesis in *Gata6*-deficient mice (Pierre et al., 2009), GATA4, and GATA6 negatively regulate Hh signaling in the development of the pancreas (Xuan and Sussel, 2016). Similarly, GATA6 antagonizes Wnt signaling in lung development, (Singh et al., 2014), but in *Xenopus Wnt11b* rescues cardiac differentiation resulting from the loss of GATA4 and GATA6 (Afouda and Hoppler, 2011).

In addition to these GATA6 connections, considerable crosstalk between the individual components of the Wnt and Hh networks is known (Song et al., 2015; Wehner and Weidinger, 2015; Jiang, 2017), which is not surprising given the similarities between the mechanisms of the two pathways, and their evolutionary relationships (Nusse, 2003). With evidence for an involvement of both pathways in the differentiation of F9 cells into PrE, we decided to explore the crosstalk that might exist between the Hh and the Wnt pathway. We found that RA-induced *Ihh* expression, Hh pathway activation as evident from the increase in Gli reporter activity, and increased expression of Hh target genes including *Gli1* and *Ptch2*. *Gata6* overexpression in F9 cells also induced *Ihh*, activated a Gli reporter and increased the expression of Hh target genes *Ptch1* and *Ptch2*. The SMO agonist SAG also stimulated the Gli reporter, but had no effect on β-catenin-dependent TCF-LEF reporter activity, and unlike RA or *Gata6* overexpression, it failed to differentiate F9 cells. In contrast, the SMO antagonist cyclopamine (Cyc) not only attenuated the RA-induced increase in Gli reporter activity and F9 differentiation, but surprisingly it reduced TCF-LEF reporter activity. Finally, results showing the inhibition of GSK3 with BIO increased TCF-LEF and Gli reporter activities suggesting the regulation of GSK3 activity as the potential node where crosstalk exists between the Hh and Wnt pathways.

## Materials and methods

### Cell culture, transfection, and treatment

Mouse F9 teratocarcinoma cells (ATCC) were cultured in Dulbecco's Modified Eagle's Medium (DMEM; Lonza) enriched with 10% fetal bovine serum (FBS; Gibco) and 1% penicillin-streptomycin antibiotic (PS; Lonza). The cells were seeded in tissue culture (TC) treated 60 mm plates (BD Falcon) for subsequent protein and RNA isolation. When cells were ~40% confluent they were treated with either 10^−7^ M retinoic acid (RA; Sigma) overnight, and then subsequently with 10 μM Cyclopamine (Cyc; EMD Millipore) for luciferase assays or treated with either 5 or 10 μM Cyc simultaneously with 10^−7^ M RA for immunoblot and RT-qPCR analysis. To activate Hh signaling cells were treated with either 5 or 10 nM Smoothened agonist (SAG; EMD Millipore). To inhibit GSK3, cells were treated with either 5 or 10 nM 6-Bromoindirubin-3′-oxime (BIO, Sigma-Aldrich). As a vehicle control, cells were treated with 0.05% Dimethyl Sulfoxide (DMSO). All cells were incubated at 37°C and 5% CO_2_ for a total of 96 h before immunoblot analysis, collected every 24 h for qRT-PCR analysis, or collected after 48 h for luciferase assays.

Cells were transfected with an empty vector or pcDNA3.1-*Gata6* (a gift from Dr. M. Jaconi, University of Geneva) for overexpression studies, and pGL3-*BARL* (Promega) or pGL3*-Gli* (a gift from Philip Beachy, Stanford University) with pRL-*TK* (Promega) constructs for luciferase assays using Lipofectamine2000 according to the manufacturer's recommendations (ThermoFisher Scientific). Briefly, 10 μL of Lipofectamine2000 was mixed with a total of 4 μg of expression constructs to transfect cells grown to 60% confluence in 35 mm TC treated plates (BD Falcon); for co-transfection experiments, equal amounts of each construct were used with Lipofectamine2000 to DNA ratio of 10:4. Transfected cells were passaged 24 h later into 60 mm TC plates. Transfected cells were treated at the chemical concentrations described above. In overexpression studies pcDNA3.1 transfected cells were treated with either 0.05% DMSO or 10^−7^ M RA, and were collected with pcDNA-*Gata6* transfected cells after 96 h.

### Quantitative reverse transcription polymerase chain reaction

To determine relative steady-state mRNA levels, total RNA was isolated from F9 cells at 24, 48, 72, and 96 h post-treatment/transfection using the RNeasy Mini Kit (Qiagen). RNA was reverse transcribed into first strand cDNA using the High-Capacity cDNA Reverse Transcription Kit (Applied Biosystems). Primers were designed to amplify the mouse *Shh, Dhh, Ihh, Wnt6, c-Myc, Ccnd1, Dkk1, Dab2, Gata6, Smoothened, Patched1, Patched2, Sufu, Gli1, Gli2*, and *Gli3* nucleotide sequences (Supplementary Table 1). Primers to the constitutively expressed ribosomal gene *L14* were used as controls. qRT-PCR was conducted under the following reaction conditions: 500 nM of each reverse and forward primer, SensiFAST SYBR Mix (FroggaBio), and 1 μL of cDNA template. Samples were analyzed with the CFX Connect Real-Time PCR Detection System (Bio-Rad) using the comparative cycle threshold (2^−ΔΔCt^) method, where relative expression values were obtained from steady-state mRNA levels normalized to *L14* mRNA. Relative expression values were subsequently normalized to DMSO/control plasmid treatment(s) to determine fold change.

### Immunofluorescence and confocal analysis

Cells were fixed on glass-bottom 12-well plates (MatTek) with 4% paraformaldehyde (Electron Microscopy Sciences), permeabilized with 0.1% Triton X-100, and blocked in 1% bovine serum albumin. Cells were incubated overnight at 4°C with TROMA-1 antibody (1:50; Developmental Studies Hybridoma Bank) and Alexa488-conjugated anti-rat antibody (ThermoScientific) for 2 h, followed by DAPI (Molecular Probes) and Vectashield antifade reagent. All reagents were diluted in phosphate buffered saline. Cells were examined using a Zeiss LSM 5 Duo Vario Microscope with both ZEN and Image-Pro Premier 3D software.

### Immunoblot analysis

Cells were lysed in 300 μL of 2% sodium dodecyl sulfate buffer containing 62.5 mM Tris-HCL pH 6.8, 10% glycerol, 5% Mercapto-2-ethanol, and 1X Halt Protease Inhibitor Cocktail (Thermo Scientific). Protein concentrations were measured using a Bradford assay (Bio-Rad), and 10–30 μg of total protein were separated on denaturing 10% polyacrylamide gels and transferred to Immunoblot PVDF membrane (BioRad). Membranes were incubated in Tris-buffered saline with 0.1% Tween-20 containing 5% skim milk for 1 h at room temperature. Blots were probed with primary antibodies to TROMA-1 (1:50), DAB2 (1:10,000; Santa Cruz) and β-actin (1:10,000; Pierce) overnight at 4°C. After extensive washes, membranes were probed with the appropriate HRP-conjugated secondary antibodies (1:1,000), and washed extensively with Tris-buffered saline. Signals were detected using the Luminata Classico Western HRP Substrate (Millipore), and imaged using a Chemi Doc Touch Imaging System (Bio-Rad).

### TCF/LEF and Gli reporter assays

Cells transfected with either pGL3-*BARL* (β-catenin activated reporter luciferase) or pGL3-*Gli* were treated with 0.05% DMSO (vehicle control), 10^−7^ M RA, 10 μM Cyc alone, 5 or 10 nM BIO, 5 or 10 nM SAG, or 10 μM Cyc with 10^−7^ M RA, were prepared 48 h after treatment for luciferase assays using the Dual Luciferase Assay Kit as per manufacturer's instructions (Promega). Cells transfected with either pGL3-*BARL* or pGL3-*Gli*, and then co-transfected with pcDNA3.1 (empty vector control) or pcDNA3.1-*Gata6* were also prepared 48 h post-transfection for luciferase assays. Cells were co-transfected with pRL-*TK* to normalize luciferase levels. Luciferase expression was quantified using the GloMax System (Promega).

### Statistical analysis

Data from all experiments are representative of at least three independent biological replicates performed on separate occasions. Data comparisons between the control and treated groups were performed using a one-way ANOVA with Tukey's honest significant difference (HSD) post-hoc analysis or Student's *t*-test (SPSS Statistics for Windows Version 19.0, IBM Crop. Released 2010, Armonk, NY). *P*-values were considered statistically significant at the 0.05 level.

## Results

### Genes encoding Hh components are altered in response to retinoic acid

**Figure 1:**
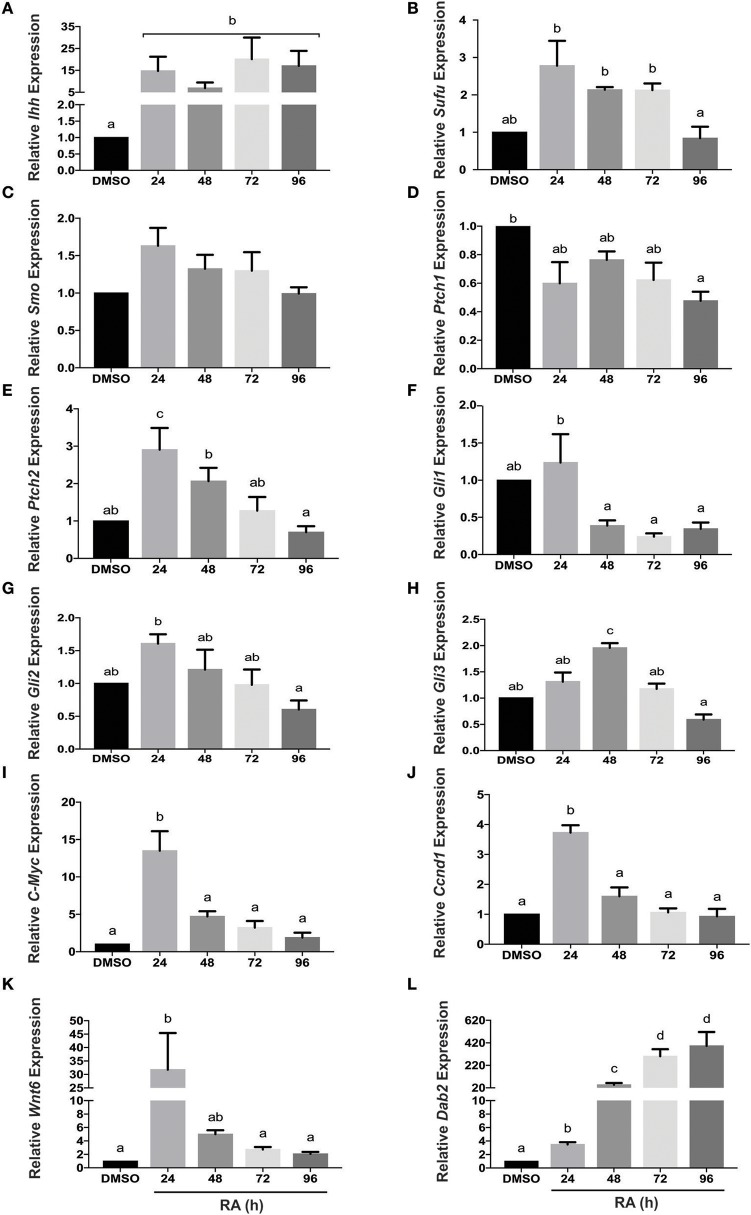
*Hh* pathway component and target gene mRNA levels in response to RA-induced PrE differentiation. Total RNA was extracted from F9 cells treated with 10^−7^ M RA to induce PrE at 24, 48, 72, and 96 h post-treatment, and qRT-PCR was performed to determine the expression of: **(A)** *Indian hedgehog* (*Ihh*), **(B)** *Sufu*, **(C)** *Smoothened* (*Smo*), **(D)** *Patched-1* (*Ptch1*), **(E)** *Patched-2* (*Ptch2*) **(F–H)** *Gli1, Gli2, and Gli3*, **(I)** *c-Myc*, **(J)** *Ccnd1*, **(K)** *Wnt6*, and *Dab2* **(L)**. Data are representative of three independent experiments ± SEM. Letters indicate significant difference (*p* < 0.05) from the DMSO control as tested by One-Way ANOVA followed by a Tukey test relative to the constitutively expressed *L14* gene (2^−ΔΔCt^).

F9 cells treated with RA differentiate into PrE, and this is accompanied by an increase in *Ihh* expression (Becker et al., 1997). Two other Hh genes are expressed in mouse and for this reason the expression profiles of *Ihh, Shh*, and *Dhh* were examined in undifferentiated F9 cells and those differentiated to PrE. Total RNA was collected from cells treated with DMSO (vehicle control) or 10^−7^ M RA from 24 to 96 h post-treatment. Results showed that by 24 h, RA caused a significant increase in *Ihh* expression relative to DMSO controls (Figure 1A). In contrast, the relatively high Ct values for *Shh* and *Dhh* would indicate that there were negligible changes in the levels of mRNA due to RA treatment (data not shown). Thus, *Ihh* was an RA-responsive gene and transcript levels increased during PrE differentiation, along with known PrE markers *Gata6* (Supplementary Figure 2), *Wnt6* (Figure 1K). The levels of *Dab2* also increased (Figure 1L), which is significant as it is a Wnt target gene (Railo et al., 2009) that encodes a negative regulator of canonical β-catenin signaling in PrE (Golenia et al., 2017). Having identified *Ihh* as a candidate involved in RA-induced differentiation, we examined the expression of Hh pathway components including *Sufu, Smo, Ptch1, Ptch2, Gli1, Gli2*, and *Gli3*. *Sufu* levels were significantly higher than the DMSO control for the first 72 h of RA treatment (Figure 1B), however, no significant changes were detected in *Smo* expression (Figure 1C). Expression of *Ptch1*, a Hh target gene, also did not change significantly in response to RA (Figure 1D), whereas *Ptch2* expression, also a Hh target gene, increased significantly with RA after 24 h post-treatment (Figure 1E). *Gli1*, another Hh target gene, was thoroughly interrogated as its expression was expected to increase because of activated Hh signaling. Despite the modest increase at 24 h, levels were not significantly different from DMSO, and levels dropped significantly after the 24 h time point (Figure 1F). While no significant differences in *Gli2* expression relative to DMSO were observed at any time point (Figure 1G), *Gli3* expression was found to increase significantly with RA after 48 h (Figure 1H). Confirmation of active Wnt signaling in response to RA came from examining the expression of the Wnt target genes *c-Myc* and *Ccnd1*, both of which whose levels increased significantly at 24 h post treatment (Figures 1I,J, respectively). Active Wnt signaling was also confirmed as evident by the RA-induced, significant increase in expression of another Wnt target gene *Dickkopf-1* (*Dkk1*) that encodes a negative regulator of Wnt signaling (Supplementary Figure 3). This evidence for the RA-responsive increase in *Ihh*, and some, but not all, of the Hh signaling components prompted us to examine *Ihh* in more detail.

### GATA6 induces *Ihh* and activates Hh signaling during PrE formation

**Figure 2:**
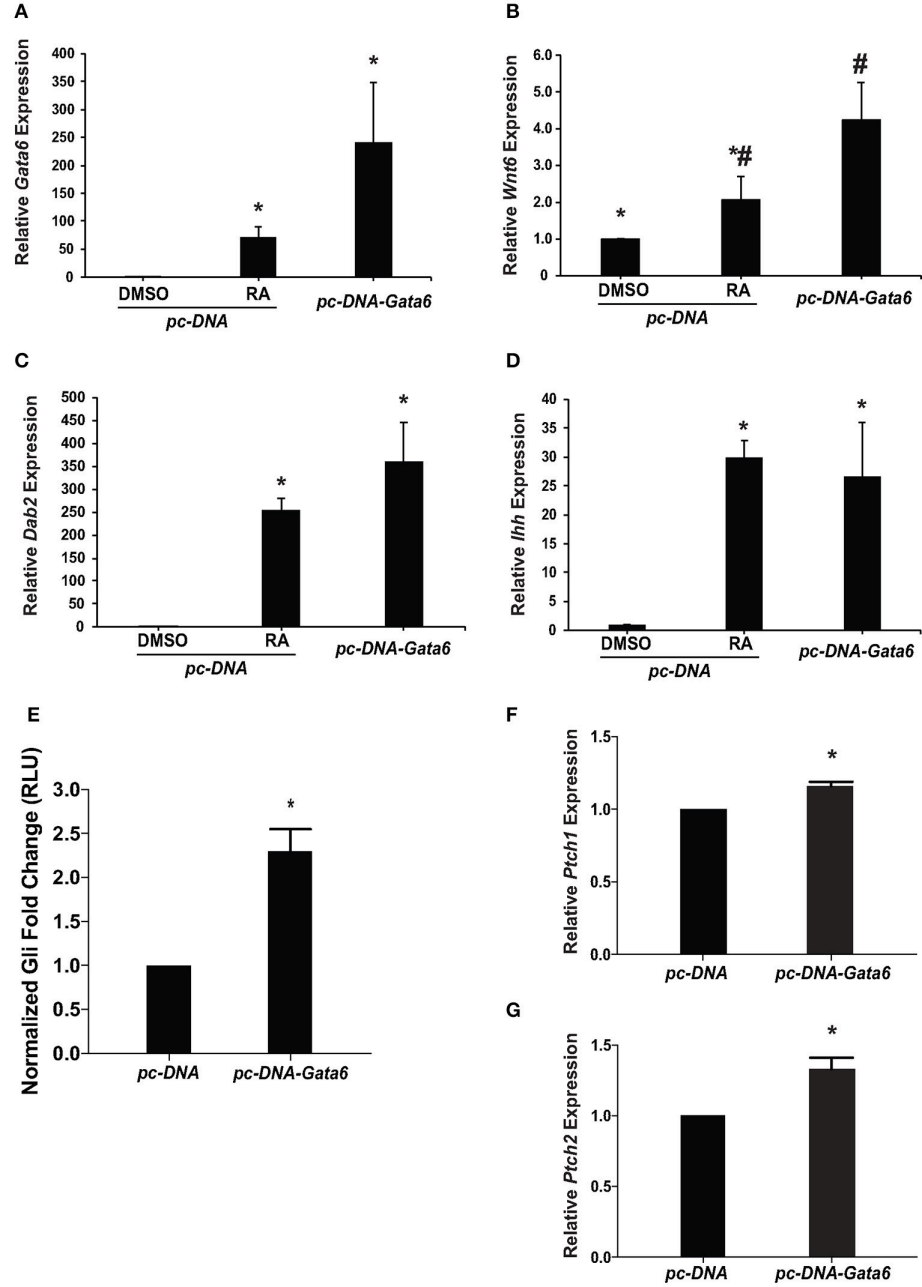
Overexpression of *Gata6* up-regulates *Ihh* expression, increases *Gli* reporter activity, and induces Hh target genes. F9 cells were transfected with a pcDNA3.1 empty vector (control) or pcDNA3.1-*Gata6*. Total RNA was collected after 96 h and was analyzed by qRT-PCR to determine the expression of: **(A)** *Gata6*, **(B)** *Wnt6*, **(C)** *Dab2*, and **(D)** *Ihh*. **(E)** F9 cells co-transfected with pGL3-*Gli* and either the empty vector control or pcDNA3.1-*Gata6* were collected 48 h post-transfection and lysates were processed to measure *Gli*-dependent luciferase activity. **(F)** *Ptch1* and **(G)** *Ptch2* expression was analyzed using qRT-PCR in F9 cells transfected with empty vector control or pcDNA3.1-*Gata6*. The data presented is from three independent experiments ± SEM. Symbols indicate significant difference (*p* < 0.05) from the DMSO control as tested by One-Way ANOVA followed by a Tukey test relative to *L14* (2^−ΔΔCt^). For luciferase reporter assay, the bars represent mean fold changes in relative light units (RLU) ± SEM, normalized to *Renilla* luciferase activity. ^*^p < 0.05 as tested by Student's *t*-test.

Given that GATA6 is involved in the RA induced differentiation of F9 cells to PrE (Hwang and Kelly, 2012), and with the identification of a putative GATA6 binding site in the *Ihh* promoter (Supplementary Figure 1), we sought to determine if overexpression of *Gata6* would up-regulate *Ihh* expression. To test this, F9 cells were transfected with pcDNA3.1 (empty vector control), and were treated with DMSO or 10^−7^ M RA or were transfected with pcDNA3.1-*Gata6*. Total RNA was collected 96 h post-treatment for qRT-PCR analysis with *L14, Gata6, Ihh, Wnt6*, and *Dab2* primers (Figure 2). As expected *Gata6* expression in RA-treated cells was significantly greater than DMSO-treated cells, and there was no significant difference in the levels between RA and those due to *Gata6* overexpression (Figure 2A). RA and *Gata6* overexpression significantly increased both *Wnt6* and *Dab2* expression compared to DMSO controls (Figures 2B,C, respectively), indicative of PrE differentiation (Cho et al., 1999; Zhuang et al., 2003; Krawetz and Kelly, 2008). *Gata6* overexpression also increased *Ihh* expression resulting in levels that were not statistically different from those induced by RA (Figure 2D). These results indicate that GATA6 signaling can directly or indirectly regulate the expression of *Ihh* and induce PrE, however, they do not address whether this increase in expression translates into activation of the Hh pathway.

To test for Hh pathway activation, F9 cells were co-transfected with pcDNA3.1 (empty vector control) or pcDNA3.1-*Gata6*, and pGL3-*Gli* and pRL-*TK* luciferase reporter constructs. Lysates were collected 48 h post-transfection and luciferase activity was compared between treatments. Results showed that pcDNA3.1-*Gata6* overexpression not only caused a significant increase in Gli reporter activity (Figure 2E), but also caused a significant increase in the levels of *Ptch1* and *Ptch2* (Figures 2F,G, respectively). Thus, *Gata6* overexpression served to regulate the *Ihh* gene, cause an increase in Gli-dependent transcription, and increase transcript levels of Hh responsive genes, which is accompanied by an increase in the expression of *Dab2*, a Wnt target gene and PrE marker.

### Hh signaling is required but not sufficient for PrE formation

**Figure 3:**
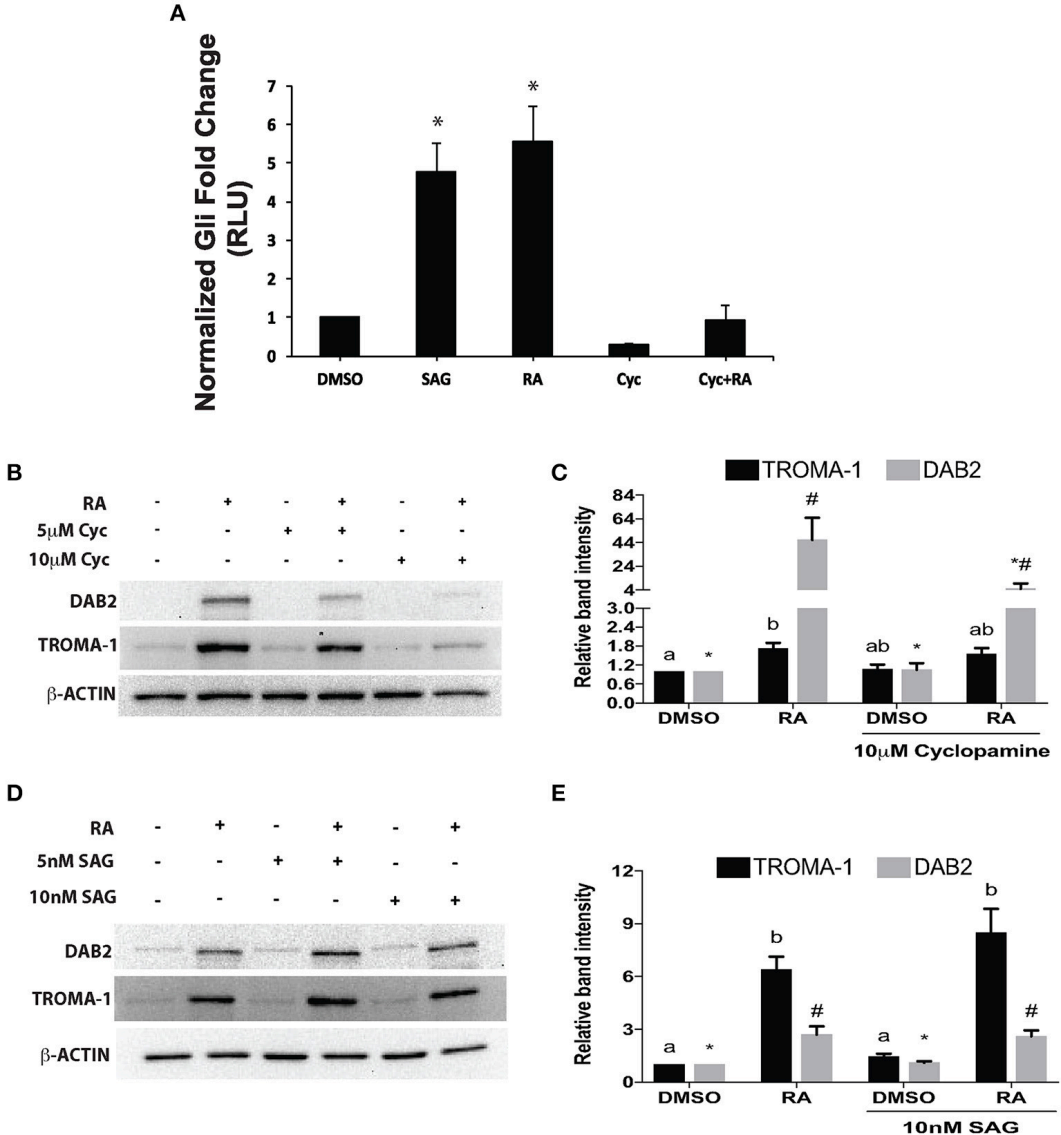
Retinoic acid increases *Gli*-mediated transcriptional activity that is necessary, but not sufficient to induce PrE. **(A)** Lysates from F9 cells co-transfected with pGL3-*Gli* and *Renilla* luciferase vector and subsequently treated with DMSO, 10 nM SAG, 10^−7^ M RA, 10 μM Cyc, or RA and Cyc, were collected 48 h after treatment. Bars represent mean fold changes in relative light units (RLU) ± SEM, normalized to *Renilla* luciferase activity. **(B)** Protein lysates were collected from F9 cells treated with DMSO, 10^−7^ M RA, 5 or 10 μM Cyc, or RA and Cyc, or **(D)** with DMSO, 10^−7^ M RA, 5 or 10 nM SAG, or RA and SAG, and then processed for immunoblot analysis with antibodies to TROMA-1 and DAB2 to detect markers of differentiation. β-actin was used as a loading control. **(C,E)** Densitometric analysis from their respective blots showing data presented from three independent experiments ± SEM. Letters and symbols indicate significant difference from the DMSO control tested by One-Way ANOVA followed by a Tukey test. ^*^p < 0.05.

Since overexpressing *Gata6* stimulated the Gli reporter, and RA induces *Gata6*, then RA should also activate the pGL3-*Gli* reporter construct. To confirm this a pGL3-*Gli* luciferase reporter and a pRL-*TK Renilla* luciferase construct were co-transfected into F9 cells, which were then subsequently treated with DMSO (vehicle control) or 10^−7^ M RA. Results showed that treatment with RA led to a significant increase in luciferase activity relative to that in the DMSO control (Figure 3A). Having established the hierarchy between RA and GATA6, we then activated the Hh pathway with SAG, a SMO agonist, and tested the luciferase activity of the Gli reporter. SAG-treated cells showed a significant increase in luciferase activity compared to DMSO controls (Figure 3A). Thus, the data indicates that upstream control of *Gata6* through RA and chemical activation of the Hh pathway are sufficient to induce Gli reporter activity. To further confirm this result, cells were treated with Cyc to block Hh signaling at the level of SMO, to determine that the RA-induced increase in Gli reporter activity was indeed due to activation of the Hh pathway. Treating F9 cells with 10 μM Cyc alone had no effect on the Gli reporter, however, 10 μM Cyc attenuated the RA response in Gli luciferase activity to levels comparable to controls cells (Figure 3A). Therefore, these results indicate that RA activates the Hh pathway in cells destined to form PrE, and the candidate ligand involved is IHH.

**Figure 4:**
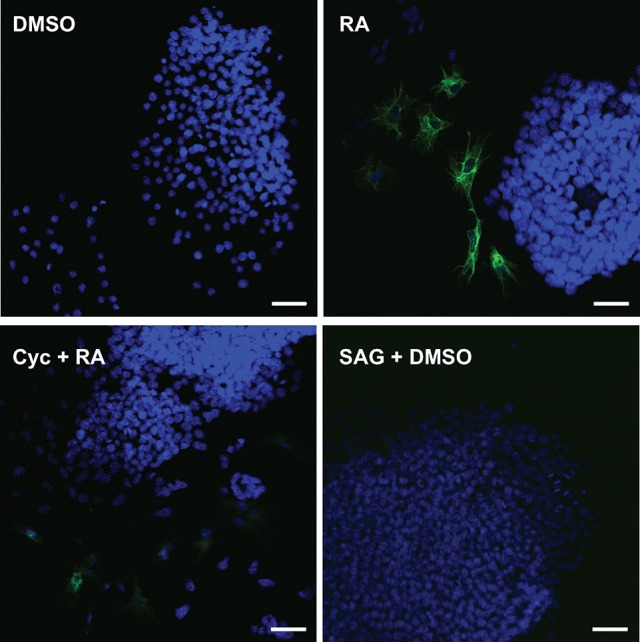
Cyclopamine blocks the assembly of KERATIN-8/18 positive filaments that form following RA treatment. F9 cells were treated with DMSO (negative control), 10^−7^ M RA (positive control), 10 μM Cyc and RA, or 10 nM SAG and DMSO. Cells were fixed at 96 h post-treatment and processed for immunocytochemistry using the TROMA-1 antibody to detect KERATIN-8/18 and counterstained with DAPI to detect nuclei. Scale bar is equal to 50 μM.

To test whether the attenuation of RA-induced increase in Gli reporter activity by Cyc (Figure 3A) had an effect on PrE differentiation, cells were treated with DMSO, 10^−7^ M RA, RA and 5 or 10 μM Cyc, or 5 or 10 μM Cyc alone, then collected after 96 h and processed for immunoblot analysis using antibodies to Disabled homolog 2 (DAB2), and TROMA-1, an antibody against KERATIN 8/18, which is a marker of PrE (Duprey et al., 1985). Results show that DAB2 was absent in DMSO controls, but was detected in RA-treated cells (Figure 3B). Cyc alone had no effect on DAB2, regardless of the concentration used, however, the intensity of the RA-induced signal decreased when cells were co-treated with 5 μM Cyc and RA, and more dramatically in cells co-treated with 10 μM Cyc and RA. TROMA-1 signals were detected in all treatment regimens, but the signal intensity was significantly increased with RA treatment (Figure 3B). Interestingly, these signals declined when cells were co-treated with RA and either concentrations of Cyc (Figure 3B). Densitometric analysis confirmed these results (Figure 3C), and confirmed that activation of the Hh pathway is necessary for RA-induced PrE. *Dkk1* expression was also examined, and while levels in controls increased significantly due to RA, they dropped when cells were co-treated with RA and Cyc (Supplementary Figure 3). Since the data pointed to active Hh signaling being required during the induction of PrE, we predicted that activation of the Hh pathway alone was sufficient to induce PrE differentiation. To test for sufficiency, F9 cells were treated with DMSO, 10^−7^ M RA, RA co-treated with 5 or 10 nM SAG, or 5 or 10 nM SAG alone. Protein lysates were collected after 96 h for immunoblot analysis for DAB2 and TROMA-1 detection (Figure 3D). Results showed a weak DAB2 signal in DMSO-treated cells, which was comparable to those detected when cells were treated with 5 or 10 nM SAG alone. The intensity of the band increased, however, when cells were treated with RA, but there was no further increase in band intensity when RA-treated cells were co-treated with the highest SAG concentration. Similar results were seen with the TROMA-1 signal, and densitometric analysis confirmed that 10 nM SAG alone had not significantly altered DAB2 or TROMA-1 levels relative to the RA-alone control (Figure 3E). Thus, despite SAG activating Gli-mediated transcription (Figure 3A), and upregulating *Gli1* and *Ptch2* expression, but not *Ptch1* (Supplementary Figure 4), the densitometric data confirmed that activating Hh signaling alone was not sufficient to induce PrE. As further proof, immunofluorescence microscopy using the TROMA-1 antibody was used to corroborate the immunoblot data. Results showed that KERATIN-8/18-positive intermediate filaments were only present in cells treated with RA alone (Figure 4). These results indicate the activation of the Hh pathway is necessary for RA-induced F9 cell differentiation; however, activating the Hh pathway alone is not sufficient for the cells to differentiate toward a PrE lineage.

### Pre differentiation involves hedgehog and canonical β-catenin pathways

**Figure 5:**
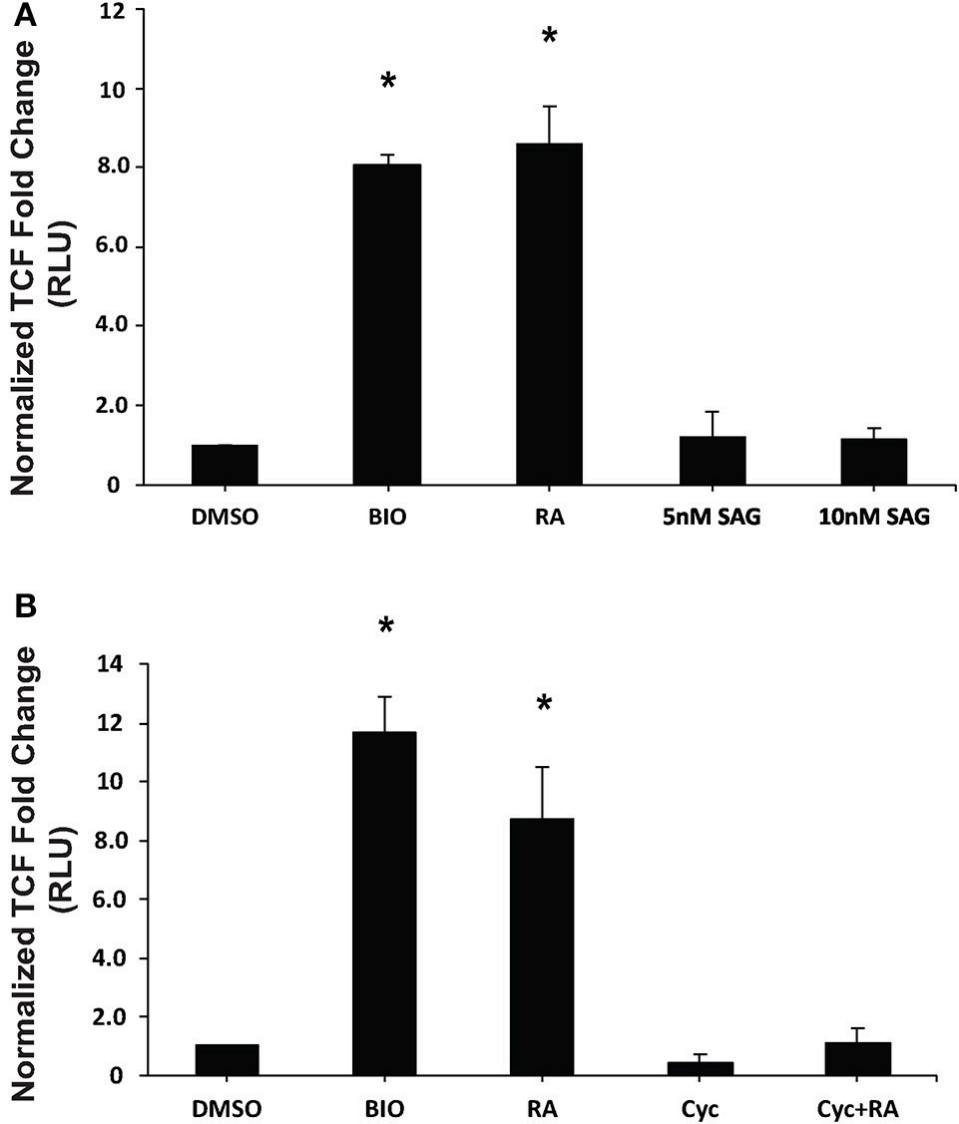
Chemical activation/inhibition of the Hedgehog pathway affects TCF/LEF transcriptional activity. F9 cells were co-transfected with pGL3-*BARL* and *Renilla* luciferase vectors and were collected 48 h after treatment with **(A)** DMSO, 10 nM BIO, 10^−7^ M RA, or 5 or 10 nM SAG, or **(B)** DMSO, 10 nM BIO, 10^−7^ M RA, 10 μM Cyc, or RA and Cyc, for luciferase assays to measure TCF-LEF activity. Bars represent mean fold changes in relative light units (RLU) ± SEM, normalized to *Renilla* luciferase activity. ^*^p < 0.05.

**Figure 6:**
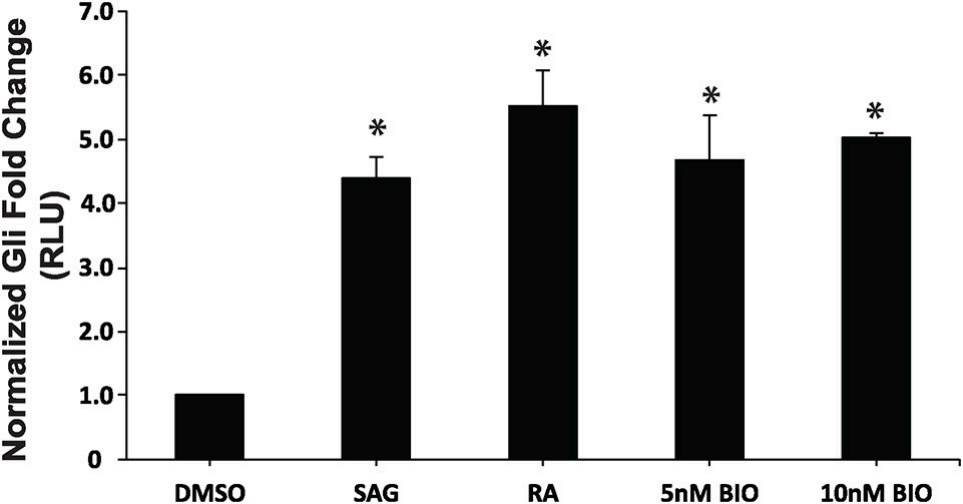
Inhibition of GSK3 activity increases *Gli*-mediated transcription. F9 cells were co-transfected with pGL3-*Gli* and *Renilla* luciferase vectors, and were collect 48 h after treatment with DMSO, 10 nM SAG, 10^−7^ M RA, 5 or 10 nM BIO. Data are representative of three independent experiments. Bars represent mean fold changes in relative light units (RLU) ± SEM, normalized to *Renilla* luciferase activity. ^*^p < 0.05.

Previous work has established that Wnt/β-catenin and Hh pathways form integrative signaling webs, however, the specifics of the crosstalk between them are poorly understood. Given this information, and the fact that activation of Hh was required, but not sufficient to induce PrE in a process requiring Wnt/β-catenin signaling (Liu et al., 2002), we next wanted to examine the relationship between the two pathways in PrE differentiation. Cells were transfected with either pGL3-*Gli* or pGL3-*BARL* reporter constructs, the latter as a readout of active Wnt signaling, and with a *Renilla* luciferase construct to normalize the data. To determine if the Hh pathway can signal to the Wnt pathway, F9 cells were first transfected with the pGL3-*BARL* construct and treated with 10 nM BIO, a GSK3 inhibitor, or with DMSO, 10^−7^ M RA, or 5 or 10 nM SAG. Results showed that the 5 and 10 nM concentrations of SAG were not sufficient to significantly activate pGL3-*BARL* activity (Figure 5A). RA, as expected, caused a significant increase in BARL activity relative to the control, and these results were not significantly different from those seen from BIO-treated cells (Figures 5A,B). The experiment was repeated, however, in this case the Hh pathway was chemically inhibited using 10 μM Cyc. Given that Cyc treatment with RA significantly decreased the levels of two Wnt signaling components, DAB2 (Figure 3B) and *Dkk1* (Supplementary Figure 3), we expected Cyc would affect the activity of the pGL3-*BARL* reporter. However, results showed that when cells were co-treated with 10 μM Cyc and RA there was no significant difference in luciferase activity compared to control treatments (Figure 5B). This reduction contrasted the significant increase in reporter activity caused by RA alone (Figure 5B). Together, these results revealed that while active Hh signaling alone is not sufficient to induce markers of PrE differentiation or increase TCF/LEF transcriptional activity, the ability of Cyc to attenuate RA-induced TCF reporter activity and PrE differentiation, implicates active Hh signaling as being involved. Having identified a link between Hh and Wnt/β-catenin, one possible candidate serving as a node that could inhibit both pathways is GSK3β (Jia et al., 2002). If this is true in F9 cells, then inhibiting GSK3β activity should also stimulate the Gli reporter. To test this, F9 cells were transfected with the pGL3-*Gli* reporter and then treated with DMSO, 10 nM SAG (positive control), 10^−7^ M RA (positive control), or with 5 or 10 nM BIO (Figure 6). Results showed that the SAG treatment caused a significant increase in Gli reporter activity that was not significantly different from cells treated with RA. Interestingly, the 5 and 10 nM BIO treatments caused an increase, both of which were significantly different from DMSO controls, and comparable to the SAG and RA results. Thus, while activating the Hh pathway had no detectable effect on β-catenin-TCF/LEF-dependent transcription, inhibiting GSK3β increases the transcriptional activity of both pathways, and specifically the activity of Gli-mediated transcription.

## Discussion

Signaling networks play major roles in regulating developmental processes and one of the pathways critical in the formation of the embryo involves the morphogen Hh. Although many studies have documented the involvement of Hh in development, little is known of its role in ExE differentiation. By utilizing the F9 teratocarcinoma cell line, which models ExE differentiation to PrE through chemical treatment with RA, this study has extended earlier reports showing IHH implicated in these events (Becker et al., 1997). That no detectable changes in the expression of the other two *Hh* genes encoding the Sonic and Desert isoforms, would indicate that *Ihh* is the candidate gene up-regulated during differentiation in response to RA (Figure 1), which corroborates the study noted above (Becker et al., 1997). We also examined changes in the expression of the Hh pathway components; those that are targets of Hh signaling activation, *Gli1, Ptch1*, and *Ptch2* (Falkenstein and Vokes, 2014), and those that are regulated through an, as of yet, unknown pathway. Activation of Hh target genes occurs primarily because of the post-translational increase of the GLI1/GLI2 activator and/or reduction of the GLI3 repressor. In this study significant, albeit few, changes were detected in *Gli1* and *Gli3* expression in response to RA (Figures 1F,H, respectively), the former being a known Hh target gene (Lee et al., 1997). The increase in *Ptch2* expression following RA treatment (Figure 1E) was expected, as this gene is also commonly used as an indicator of Hh signaling (Rohatgi et al., 2009). That the mRNA expression of some of the Hh components was not affected by RA, would suggest that the regulation of these components during the differentiation of PrE is due to post-translational modifications. This supposition is currently being tested, but it may be difficult to come to conclusions given the range interactions with other proteins, or the GLI transcription factor activator to repressor ratio being more representative of active/inactive signaling than the levels of the proteins themselves.

RA treatment of F9 cells regulates a plethora of genes (Eifert et al., 2006; Su and Gudas, 2008; Kelly and Gatie, 2017) including *Gata6*, which as described earlier plays an integral role in patterning the extraembryonic and embryonic endoderm (Cai et al., 2008; Kelly and Drysdale, 2015). In F9 cells *Gata6* expression increases in response to RA, and when translated it binds to the *Wnt6* promoter (Hwang and Kelly, 2012). *In silico* analysis revealed the *Ihh* promoter contains a putative GATA6 binding site (Supplementary Figure 1), suggesting that the gene is also regulated by this transcription factor. In support, studies have shown that IHH rescues definitive hematopoiesis in *Gata4* and *Gata6*-defecient murine embryoid bodies (Pierre et al., 2009). That *Ihh* is up-regulated in response to RA (Figure 1A), RA up-regulates *Gata6* (Hwang and Kelly, 2012), and the existence of a putative GATA6 binding site in the *Ihh* promoter (Supplementary Figure 1) prompted us to test for a possible link between these players during the differentiation of PrE. F9 cells were transfected with a *Gata6* expression vector and qRT-PCR analysis showed the 250-fold increase in *Gata6* expression (Figure 2A) caused a concomitant increase in the expression of *Wnt6, Dab2* and *Ihh* relative to the DMSO control (Figures 2B–D). Based on these increases in *Wnt6* and *Dab2*, which we have shown previously to accompany PrE formation (Krawetz and Kelly, 2008; Hwang and Kelly, 2012; Golenia et al., 2017), subsequent experiments were done to test if *Gata6* expression would activate the Hh pathway. Results showing *Gata6* overexpression led to a significant increase in Gli reporter activity relative to the empty vector (Figure 2E), and caused significant increases in *Ptch1* and *Ptch2* expression (Figures 2F,G, respectively) placed GATA6 in a signaling hierarchy upstream of *Wnt6* (Hwang and Kelly, 2012) and Hh signaling in PrE formation. While this study is the first to show the relationship between GATA6 and IHH signaling in this early developmental event we should note that others have reported the converse later in the development of other systems, where GATA6 represses *Shh* expression (Kozhemyakina et al., 2014; Xuan and Sussel, 2016). Nevertheless, having established that a hierarchy exists between RA, GATA6, WNT6, and IHH, subsequent tests were performed to determine if inhibiting or activating the Hh pathway would affect PrE formation.

The Smoothened agonist (SAG) and antagonist cyclopamine (Cyc) are a well-known activator and inhibitor, respectively, of the Hh pathway, and initial experiments demonstrated that both affected the Gli reporter in F9 cells (Figure 3A). These results were encouraging and suggested that the Hh pathway was sufficient and/or necessary for PrE formation. F9 and P19 embryonal carcinoma cells respond to RA by increasing the levels of DAB2 (Cho et al., 1999; Golenia et al., 2017), and KERATIN 8/18 (Duprey et al., 1985; Krawetz et al., 2011), which we confirmed by immunoblot analysis (Figures 3B,C). Cyc dramatically reduced the levels of these markers induced by RA, which suggested that active Hh signaling must promote PrE differentiation. Although, the literature is sparse in regards to Cyc and its effects on *Dab2* gene expression, there is evidence for activation of the Hh pathway with purmorphamine, another Smo agonist, showing reduced expression of the *Dab2* marker in developing zebrafish venous cells (Williams et al., 2010). Since the Wnt and Hh pathways share numerous target genes including those encoding *n-Myc, Follistatin, Jagged2*, and *Snail* (Katoh and Katoh, 2009), the Cyc-dependent decrease in DAB2 levels induced by RA suggested that *Dab2* could be another Wnt-Hh target gene. Unfortunately, SAG treatment of F9 cells had no apparent positive or negative effect either alone or in combination with RA on DAB2 (Figures 3D,E), which would indicate that it is not a direct target of Hh. The same appears to be true for KERATIN 8/18 as detected with the TROMA-1 antibody (Figure 4), but again given the paucity in the literature, contradictory results have shown increases in KERATIN 8/18 expression due to Shh signaling in mouse and human cells (Das et al., 2013; Liang et al., 2016). Thus, despite the fact that PrE markers decreased when the Hh pathway was blocked in F9 cells, it is difficult to provide a compelling explanation for why these markers did not appear in the presence of active Hh signaling. One possibility, however, is that the major contribution of Hh signaling is not the direct activation of genes required to induce markers of PrE differentiation, but rather the post-translational activation or inhibition of some other component(s), possibly the GLI transcription factors, which then act to promote expression of Wnt target genes required to induce PrE. This suggests the necessity of Hh signaling in PrE differentiation, however this signaling pathway is not sufficient. Previous studies support this idea as β-catenin can interact with GLI1 (Zinke et al., 2015), and GLI3R (Ulloa et al., 2007) in a complex that would require the activation of both Wnt and Hh signaling to induce F9 cell differentiation. To investigate this further F9 cells were transfected with a Wnt/β-catenin dependent pBARL reporter and its activity was measured in response to active (SAG) or inactive (Cyc) Hh signaling (Figure 5). While RA and BIO, the latter serving to inhibit GSK3 activity served as positive controls, SAG was unable to activate the pBARL reporter (Figure 5A). Nevertheless, the attenuation of RA-induced pBARL activity by Cyc pointed to active Hh signaling being required to cooperate positively with the Wnt/β-catenin pathway that we and others have shown to be required for F9 cells to form PrE (Liu et al., 2002; Krawetz and Kelly, 2008). Since a variety of compounds act positively or negatively on both pathways (Seke Etet et al., 2012; Distler et al., 2014; Kwon et al., 2015; Zhou et al., 2016), an alternative explanation is that Cyc may be acting in an inhibitory manner on both Wnt and Hh signaling. If so, and given the highly conserved regions in Smo and Frizzled, the WNT ligand co-receptor (Myers et al., 2013; Rana et al., 2013), it is tempting to suggest that these common areas of the proteins interact with Cyc. Unfortunately, there is no evidence for Cyc binding to Frizzled, and instead Cyc attenuation of Wnt/β-catenin signaling can be explained due to increased Hh activity acting upstream of Wnt (Mak et al., 2008; Borday et al., 2012; Qualtrough et al., 2015). In F9 cells the inability of SAG to affect the expression of Wnt inducible genes (Figures 3D, 4) or to influence the pBARL promoter (Figure 5A) would indicate that Hh is not signaling directly upstream of Wnt, and the mechanism(s) by which Cyc blocks Wnt TCF-LEF reporter activity remains to be determined.

**Figure 7:**
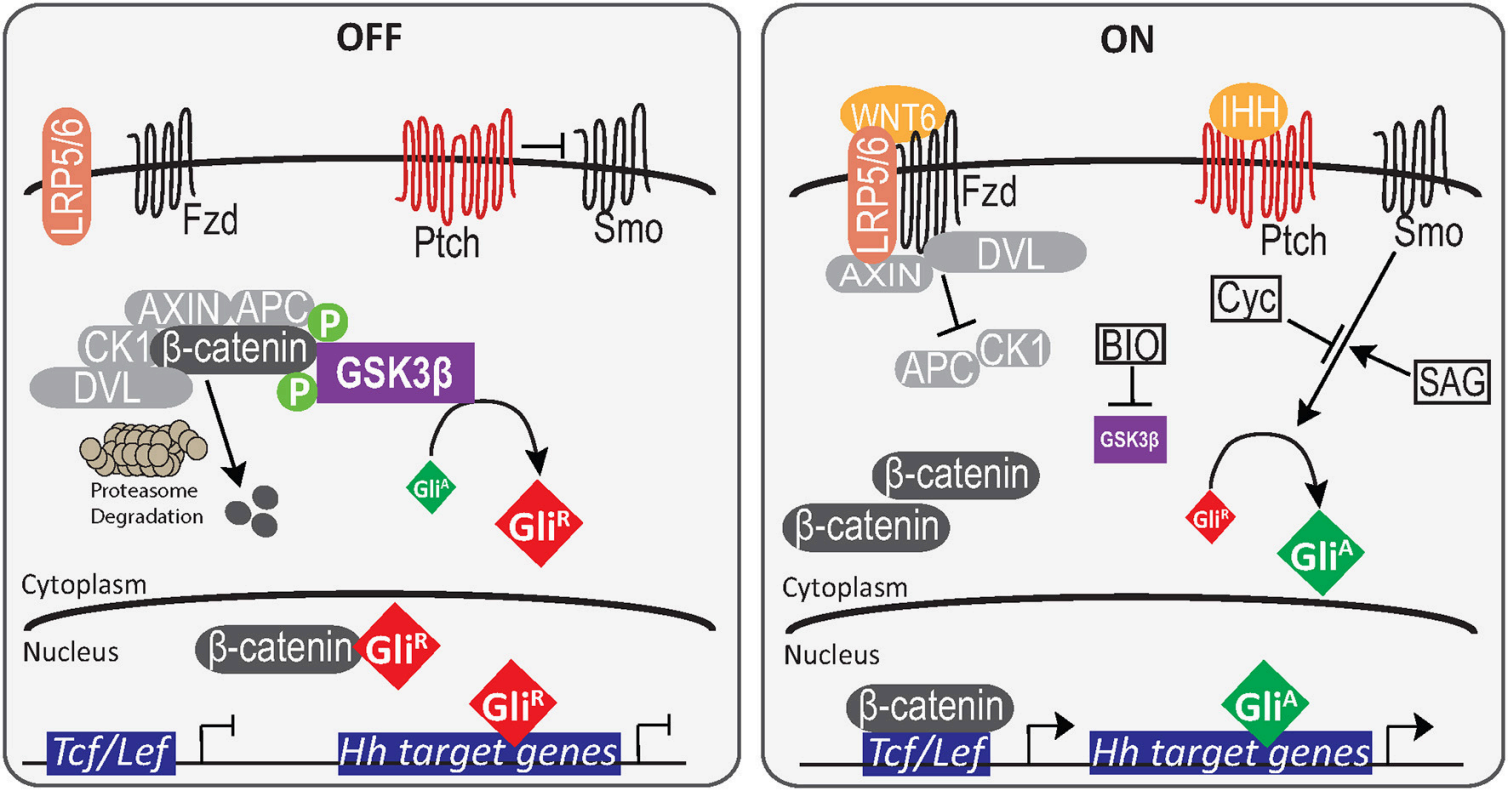
A model for primitive endoderm differentiation in F9 cells. In the absence of RA or GATA6, β-catenin is both degraded by the proteasome complex or sequestered by Gli^R^. The binding of Gli^R^ to promoter elements inhibit Gli-mediated transcription of Hh target genes. However, in the presence of RA or GATA6 (not shown), WNT6 interaction with Fzd/LRP5/6 results in the inactivation of the AXIN, APC destruction complex, and the cytoplasmic accumulation of β-catenin. The translocation of β-catenin into the nucleus and subsequent binding to TCF/LEF transcription factors promotes the upregulation of Wnt target genes. Similarly, when IHH is present it inhibits the negative regulation of PTCH on SMO, leading to the accumulation and translocation of Gli^A^ to induce Hh target genes. In the presence of the GSK inhibitor BIO, inactive GSK3β cannot phosphorylate β-catenin or promote Gli^R^ accumulation and this results in the activation of both the pBARL and Gli reporters.

Despite not having convincing evidence to explain the Cyc results, the data suggested the existence of crosstalk between Wnt/β-catenin and Hh signaling. To examine this in more detail, specifically targeting a node in regard to PrE formation, F9 cells were transfected with a Gli reporter and then treated with BIO to block GSK3 activity (Figure 6). The increase in the activity of this reporter and the TCF/LEF reporter (Figure 5) at two different concentrations of BIO suggest GSK3 as a potential node shared by the two pathways. It is interesting to note that BIO can activate *Gli1* transcription in human breast cancer cells (Das et al., 2013), and GSK3 is known to silence Hh signaling by phosphorylating the *Drosophila* GLI homolog, Cubitus interruptus (Ci) following priming by PKA (Jia et al., 2002). When Hh is absent in *Drosophila*, GSK3, PKA, and casein kinase 1 alter the activity of Ci by interacting with intermediates that enhance the proteolytic degradation of Ci into its transcriptionally repressive form (Apionishev et al., 2005; Zhang et al., 2005; Smelkinson and Kalderon, 2006). Since a similar mechanism exists in mammalian cells when Hh ligands are absent (Patel and Woodgett, 2017), BIO inactivation of GSK3 not only increases β-catenin accumulation, it would also prevent the proteolysis of GLI2/3 to their repressive forms thereby favoring the transcriptionally active form of the proteins leading to the increase in reporter activity (Figure 6). Thus, this overall regulation of Ci/GLI and Wg/Wnt by GSK3 is highly conserved (Jiang, 2017), and it appears this conservation is maintained in F9 cells. Despite having evidence that GSK3β is the node regulating Wnt and Hh signaling pathways (Figure 7), it is important to note that BIO is a non-isoform specific inhibitor of GSK3. In addition, GSK3 has been shown to interact with various signaling cascades (Taelman et al., 2010), which may regulate F9 cell differentiation in a Wnt-Hh-independent manner. Together, the data supports the view that the differentiation of F9 cells to PrE requires the cooperative activity of two signaling pathways. Specifically, the activities and regulation of Hh and Wnt target genes required for F9 differentiation to PrE is facilitated by inactivating GSK3, specifically GSK3β, which we have highlighted in a model (Figure 7). Although we have identified one potential node that is shared between these two pathways, given the complex interplay between them as evident from the Cyc results, it is likely that others exist. For that reason, we are currently evaluating other candidates that may provide additional crosstalk that fine-tunes the regulation of the Wnt and Hh pathways during extraembryonic endoderm development.

## Author contributions

GK conceived the project and along with GD. TC designed the experiments. GD, TC, LH, MG, and DS performed all the experiments. GK, GD, TC, MG, and DS analyzed the data and wrote the manuscript.

### Conflict of interest statement

The authors declare that the research was conducted in the absence of any commercial or financial relationships that could be construed as a potential conflict of interest. The reviewer CC and handling Editor declared their shared affiliation.

## Supplementary material

Supplementary Figure 1 Putative GATA6 binding site in the mouse *Ihh* promoter.

Supplementary Figure 2 Expression of *Gata6* in response to retinoic acid. Total RNA was extracted after 24, 48, 72, and 96 h from F9 cells treated with RA to induce primitive endoderm, and then subjected to qRT-PCR using primers to *Gata6*. Data are representative of three independent experiments ± SEM. Letters indicate significant difference (*p* < 0.05) from the DMSO control and relative to *L14* (2^−ΔΔCt^) as tested by One-Way ANOVA followed by a Tukey test.

Supplementary Figure 3 Cyc reduces *Dkk1* expression in RA-induced F9 cells. Total RNA was extracted after 96 h from F9 cells treated with DMSO, 10^−7^ M RA, 10 μM Cyc, or RA and Cyc, and then subjected to qRT-PCR using primers to *Dkk1*. Data are representative of three independent experiments ± SEM. Letters indicate significant difference (*p* < 0.05) from the DMSO control and relative to *L14* (2^−ΔΔCt^) as tested by One-Way ANOVA followed by a Tukey test.

Supplementary Figure 4 SAG induces the upregulation of *Gli1* and *Ptch2* in F9 cells. Total RNA was extracted after 24 h from F9 cells treated with DMSO or 10 nM SAG and then subjected to qRT-PCR using primers to **(A)** *Gli1*, **(B)** *Ptch1*, and **(C)** *Ptch2*. Data are representative of three independent experiments ± SEM. Symbols indicate significant difference from the DMSO control and relative to *L14* (2^−ΔΔCt^) as tested by Student's *t*-test. ^*^*p* < 0.05.

Supplementary Table 1 Oligonucleotide primer sequences used for qRT-PCR.
