# Supplementary figures and images for: Wnt and Hedgehog Signaling Regulate the Differentiation of F9 Cells into Extraembryonic Endoderm
Source: Front Cell Dev Biol. 2017 Oct 25;5:93. doi: 10.3389/fcell.2017.00093 (PMC5660979; doi:10.3389/fcell.2017.00093)

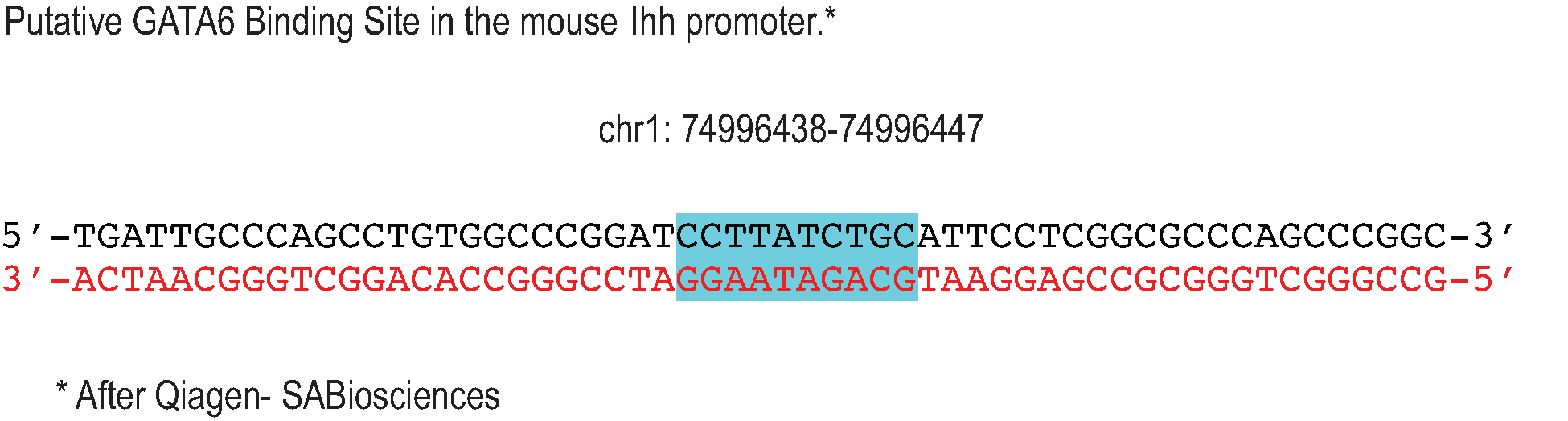

Supplement: Supplementary Figure 1 — Putative GATA6 binding site in the mouse Ihh promoter. [file Image1.TIF]

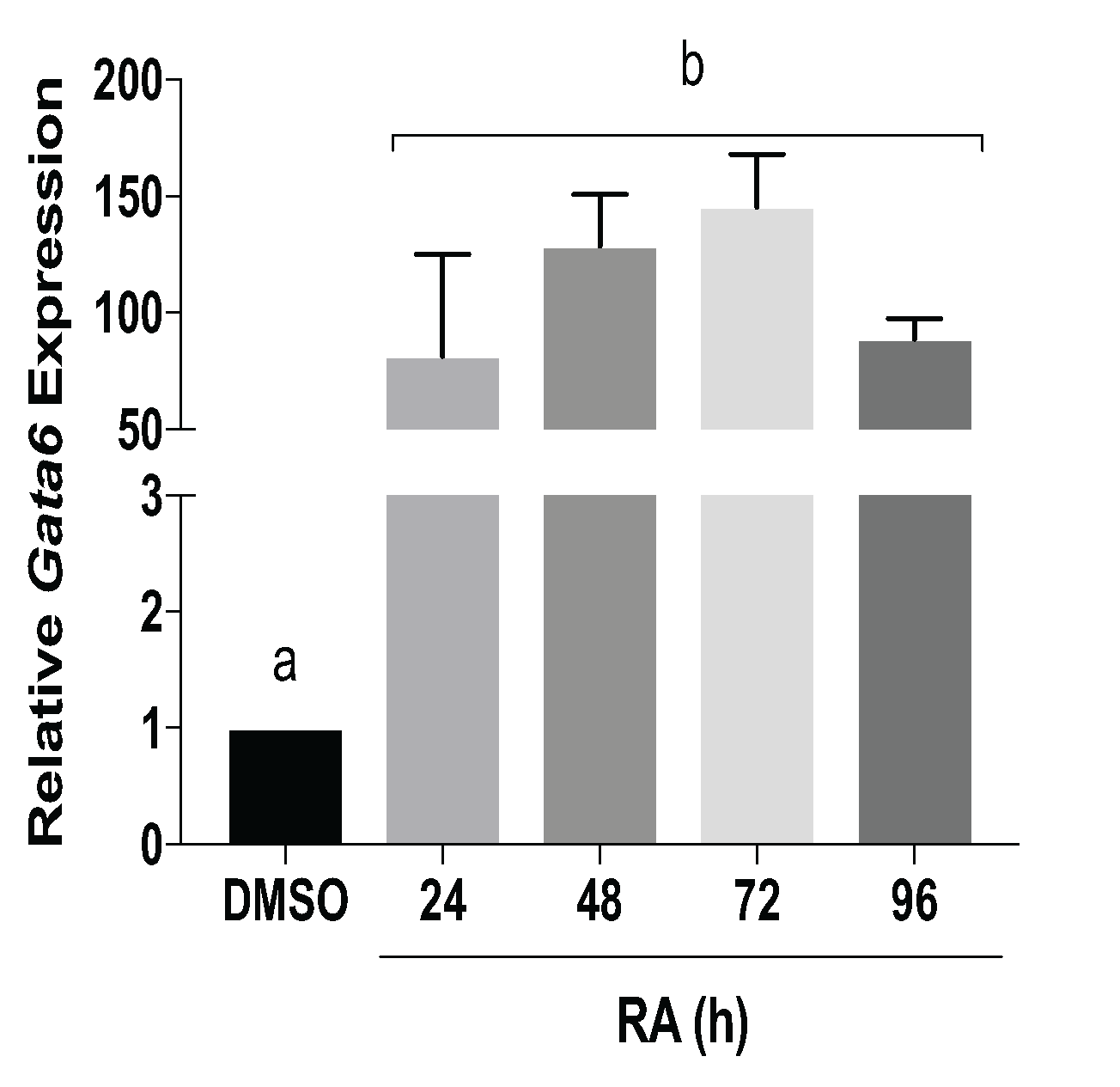

Supplement: Supplementary Figure 2 — Expression of Gata6 in response to retinoic acid. Total RNA was extracted after 24, 48, 72, and 96 h from F9 cells treated with RA to induce primitive endoderm, and then subjected to qRT-PCR using primers to Gata6. Data are representative of three independent experiments ± SEM. Letters indicate significant difference (p < 0.05) from the DMSO control and relative to L14 (2−ΔΔCt) as tested by One-Way ANOVA followed by a Tukey test. [file Image2.TIF]

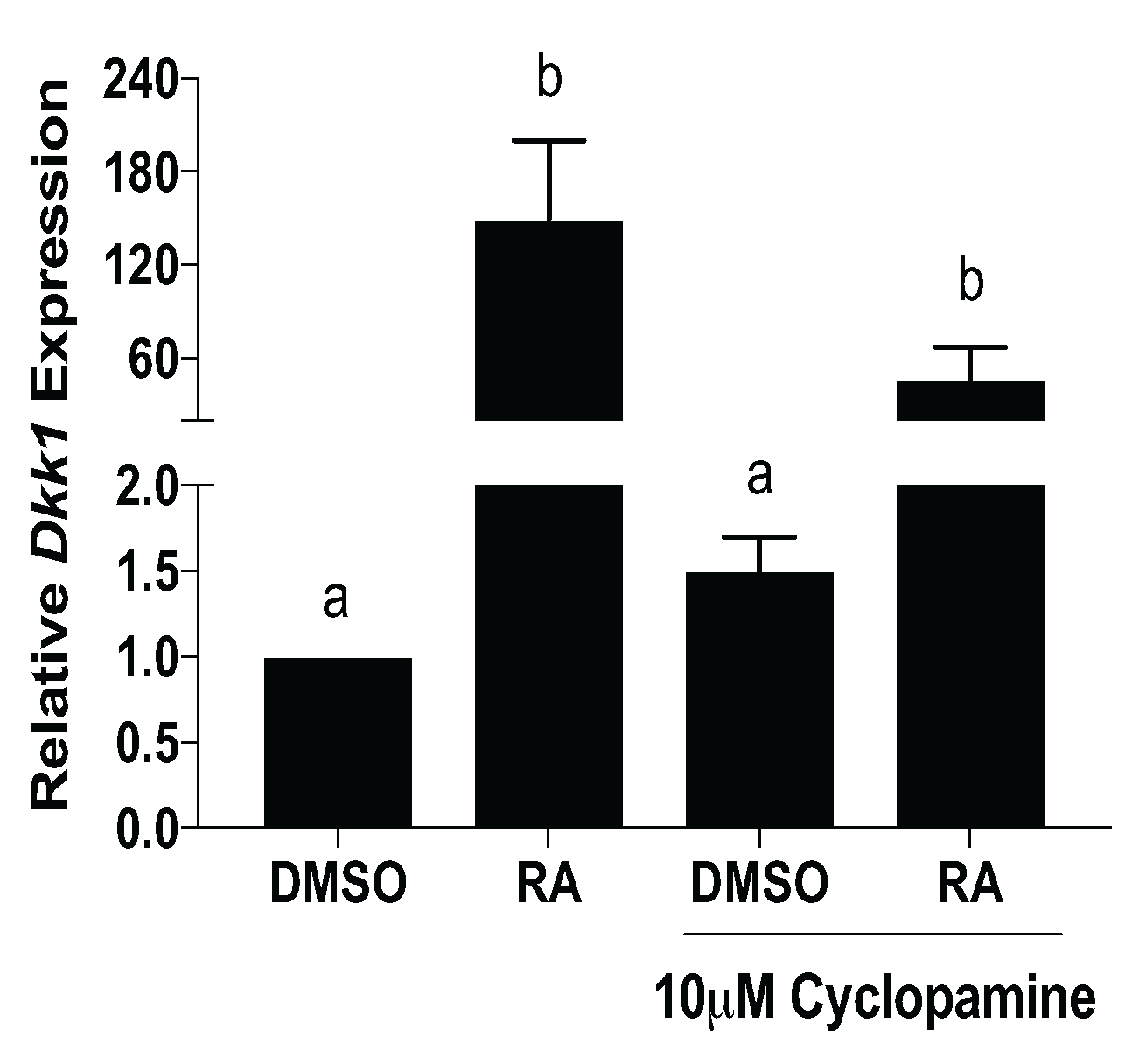

Supplement: Supplementary Figure 3 — Cyc reduces Dkk1 expression in RA-induced F9 cells. Total RNA was extracted after 96 h from F9 cells treated with DMSO, 10−7 M RA, 10 μM Cyc, or RA and Cyc, and then subjected to qRT-PCR using primers to Dkk1. Data are representative of three independent experiments ± SEM. Letters indicate significant difference (p < 0.05) from the DMSO control and relative to L14 (2−ΔΔCt) as tested by One-Way ANOVA followed by a Tukey test. [file Image3.TIF]

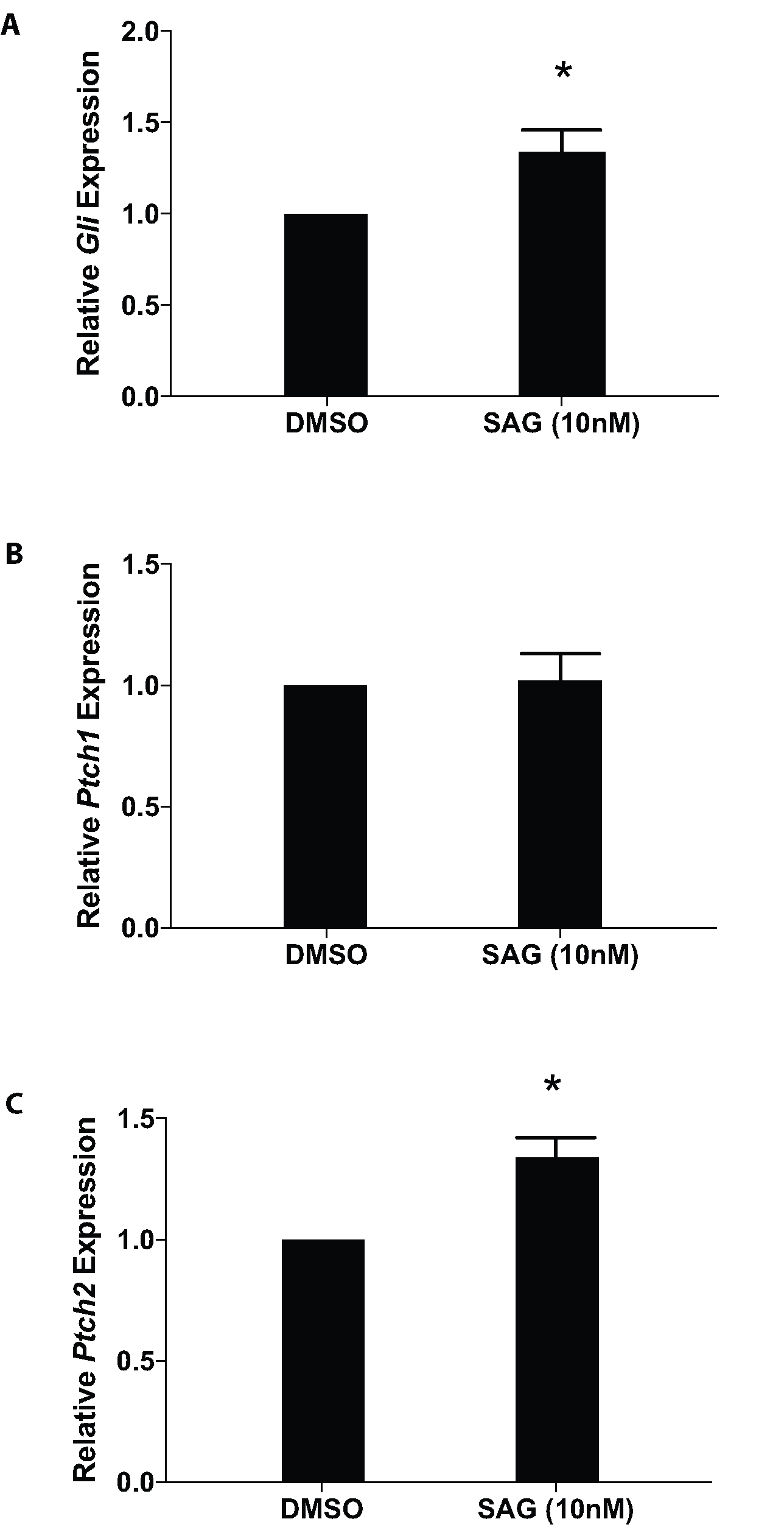

Supplement: Supplementary Figure 4 — SAG induces the upregulation of Gli1 and Ptch2 in F9 cells. Total RNA was extracted after 24 h from F9 cells treated with DMSO or 10 nM SAG and then subjected to qRT-PCR using primers to (A) Gli1, (B) Ptch1, and (C) Ptch2. Data are representative of three independent experiments ± SEM. Symbols indicate significant difference from the DMSO control and relative to L14 (2−ΔΔCt) as tested by Student's t-test. *p < 0.05. [file Image4.TIF]
