# Supplementary material for: Wnt and Hedgehog Signaling Regulate the Differentiation of F9 Cells into Extraembryonic Endoderm
Source: Front Cell Dev Biol. 2017 Oct 25;5:93. doi: 10.3389/fcell.2017.00093 (PMC5660979; doi:10.3389/fcell.2017.00093)
Supplement: Supplementary Table 1 — Oligonucleotide primer sequences used for qRT-PCR. [file Table1.DOCX]

**qRT-PCR Primers and Source doi**

| **Gene** | **Forward Primer (5’🡪3’)** | **Reverse Primer (5’🡪3’)** | **Source doi** |
| --- | --- | --- | --- |
| *Smoothened* | CCTGACTTTCTGCGTTGC | GGTTCTGACACTGAATCCG | 10.3109/10520295.2011.602645 |
| *Patched1* | TGCTGTGCCTGTGGTCATCCTGATT | CAGAGCGAGCATAGCCCTGTGGTTC | 10.1016/j.ydbio.2014.05.014 |
| *Patched2* | CCCGTGGTAATCCTCGTGGCCTCTAT | CCCGTGGTAATCCTCGTGGCCTCTAT | 10.1016/j.ydbio.2014.05.014 |
| *Gli1* | GGAAGTCCTATTCACGCCTTGA | CAACCTTCTTGCTCACACATGTAAG | 10.1186/1471-2121-9-49 |
| *Gli2* | TACCTCAACCCTGTGGATGC | CTACCAGCGAGTTGGGAGAG | 10.1242/dev.067264 |
| *Gli3* | CTGTCGGCTTAGGATCTGTTG | GCTCTTCAGCAAGTGGTTCC | 10.1038/labinvest.3700537 |
| *L14* | GGGAGAGGTGGCCTCGGACGC | GGCTGGCTTCACTCAAAGGCC | Harvard Primer Bank |
| *Shh* | TTCTGTGAAAGCAGAGAACTCC | GGGACGTAAGTCCTTCACCA | 10.1371/journal.pone.0044121 |
| *Dhh* | ACCCCGACATAATCTTCAAGGAT | GTACTCCGGGCCACATGTTC | 10.1016/j.modgep.2004.05.001 |
| *Ihh* | GACTCATTGCCTCCCAGAACTG | CCAGGTAGTAGGGTCACATTGC | 10.1002/jbmr.443 |
| *Gata6* | ATGGCGTAGAAATGCTGAGG | TGAGGTGGTCGCTTGTGTAG | 10.1128/MCB.25.7.2622-2631.2005 |
| *Wnt6* | GCAAGACTGGGGGTTCGAG | CCTGACAACCACACTGTAGGAG | Harvard Primer Bank |
| *Dab2* | GGAGCATGTAGACCATGATG | AAAGGATTTCCGAAAGGGCT | 10.1038/sj.onc.1210829 |
| *Sufu* | CGGACCCCTTGGACTATGTTA | CTTCAGACGAAACGTCAACTCA | Harvard Primer Bank |
| *Ccnd1* | GCGTACCCTGACACCAATCTC | ACTTGAAGTAAGATACGGAGGGC | 10.1038/ncomms3544 |
| *c-Myc* | CGGACACACAAGGTCTTGGAA | AGGATGTAGGCGGTGGCTTTT | 10.1038/srep22966 |
| *Dickkopf-1* | TGAAGATGAGGAGTGCGGCTC | GGCTGTGGTCAGAGGGCATG | 10.1139/bcb-2016-0150 |
